# Supplementary material for: Transcriptome Analysis Reveals Vimentin-Induced Disruption of Cell–Cell Associations Augments Breast Cancer Cell Migration
Source: Cells. 2022 Dec 13;11(24):4035. doi: 10.3390/cells11244035 (PMC9776984; doi:10.3390/cells11244035)
Supplement: Supplementary file 1 [file cells-11-04035-s001.zip › DEGs and enrichment analysis.zip!/DEGs and enrichment analysis/WT_VIMvsVector GO enrichment/UP/WT_VIMvsVector_up.GOcc_DAG.pdf]

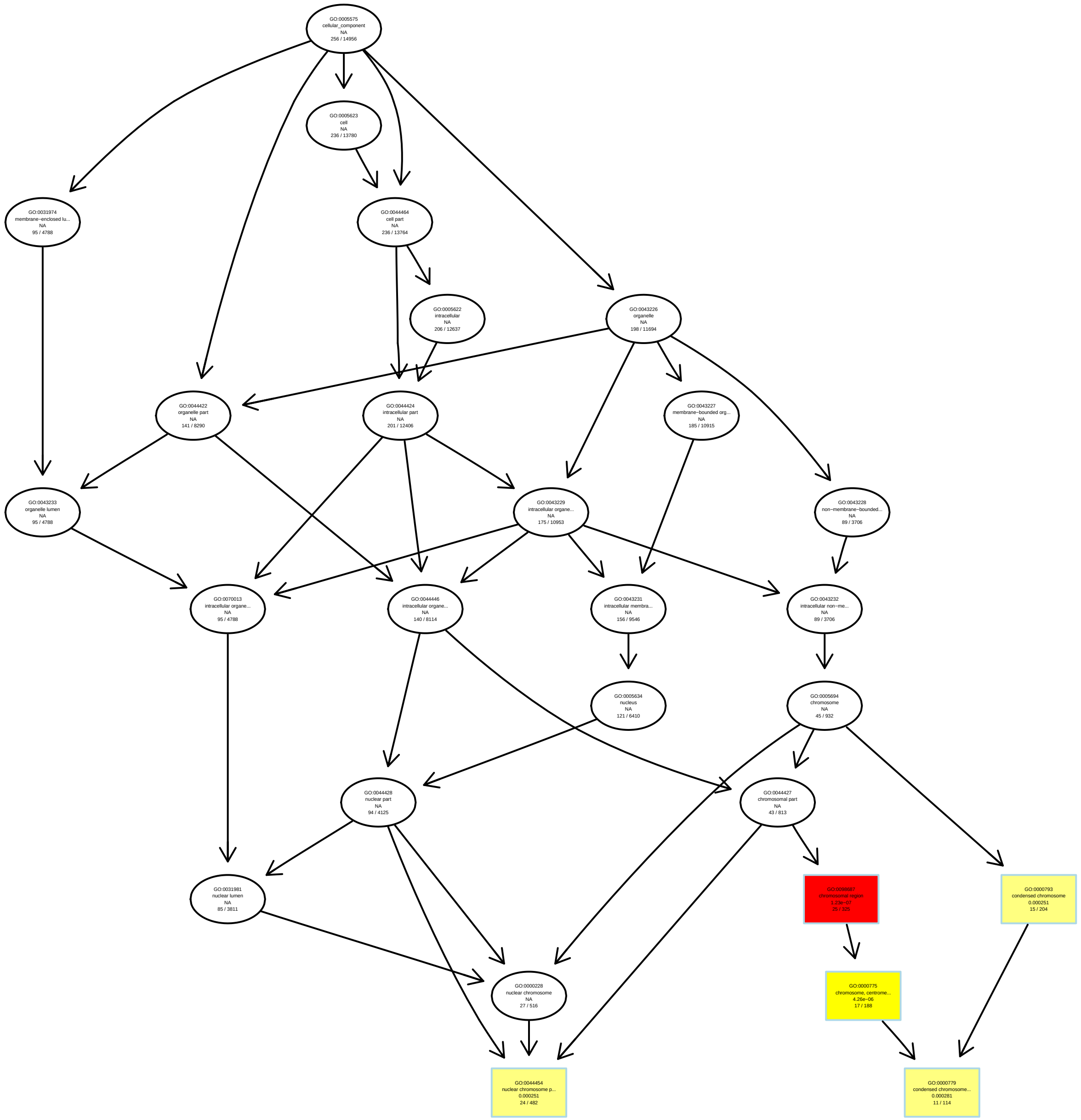
GO:0005575
cellular_component
NA
256 / 14956
GO:0005623
cell
NA
236 / 13780
GO:0031974
membrane−enclosed lu...
NA
95 / 4788
GO:0044464
cell part
NA
236 / 13764
GO:0005622
intracellular
NA
206 / 12637
GO:0043226
organelle
NA
198 / 11694
GO:0044422
organelle part
NA
141 / 8290
GO:0044424
intracellular part
NA
201 / 12406
GO:0043227
membrane−bounded org...
NA
185 / 10915
GO:0043233
organelle lumen
NA
95 / 4788
GO:0043229
intracellular organe...
NA
175 / 10953
GO:0043228
non−membrane−bounded...
NA
89 / 3706
GO:0070013
intracellular organe...
NA
95 / 4788
GO:0044446
intracellular organe...
NA
140 / 8114
GO:0043231
intracellular membra...
NA
156 / 9546
GO:0043232
intracellular non−me...
NA
89 / 3706
GO:0005634
nucleus
NA
121 / 6410
GO:0005694
chromosome
NA
45 / 932
GO:0044428
nuclear part
NA
94 / 4125
GO:0044427
chromosomal part
NA
43 / 813
GO:0031981
nuclear lumen
NA
85 / 3811
GO:0098687
chromosomal region
1.23e−07
25 / 325
GO:0000793
condensed chromosome
0.000251
15 / 204
GO:0000228
nuclear chromosome
NA
27 / 516
GO:0000775
chromosome, centrome...
4.26e−06
17 / 188
GO:0044454
nuclear chromosome p...
0.000251
24 / 482
GO:0000779
condensed chromosome...
0.000281
11 / 114
